# A novel COMMD1 mutation Thr174Met associated with elevated urinary copper and signs of enhanced apoptotic cell death in a Wilson Disease patient

**DOI:** 10.1186/1744-9081-6-33

**Published:** 2010-06-15

**Authors:** Arnab Gupta, Ishita Chattopadhyay, Shashwata Mukherjee, Mainak Sengupta, Shyamal K Das, Kunal Ray

**Affiliations:** 1 Molecular and Human Genetics Division, Indian Institute of Chemical Biology (a Unit of CSIR), Kolkata, India; 2 Movement Disorders Clinic, Bangur Institute of Neurosciences, Kolkata, India; 3 Department of Physiology, Johns Hopkins University, Baltimore, MD, USA

## Abstract

Wilson disease (WD) results from accumulation of copper and caused due to mutations in ATP7B, a copper transporting ATPase. Besides regular hepatic and neurological symptoms, WD patients occasionally manifest atypical symptoms due to unknown cause. To understand the molecular etiology of atypical WD manifestations, we screened *COMMD1*, a gene implicated in canine copper toxicosis, in 109 WD patients including those with atypical symptoms. In a patient showing apoptotic symptoms and high urinary copper surpassing normal WD levels, we identified a novel, putative mutation in *COMMD1*. Two other changes were also identified in the gene. We have examined genotype-phenotype correlation between the detected changes and the atypical presentation of the WD patient.

## Background

Wilson Disease (WD) is an autosomal recessive disorder with a frequency of 1/5,000-1/30,000 live births worldwide [1]. The disease is diagnosed on the basis of typical symptoms and conventional biochemical indicators, which include low serum concentrations of ceruloplasmin (<20 mg/dl), elevated excretion of urinary copper (>100 μg 24-hour urinary copper) and Kayser-Fleischer ring in the cornea [2]. It is caused by mutations in the *ATP7B *gene, which codes for a P-type ATPase with six copper-binding regions [3]. The protein is localized on the golgi membrane and is expressed primarily in the hepatocytes where it transports copper from cytoplasm to the bile canaliculi for excretion. In addition, the ATP7B is essential for transport of copper from the cytosol into the lumen of trans-Golgi network, where copper is utilized for biosynthetic incorporation into secretory copper-requiring enzyme, ceruloplasmin [4]. Mutations in *ATP7B *impair copper export, resulting in accumulation of the same mainly in the liver and brain and also in cornea. Clinical manifestation varies from patient to patient in terms of age of onset, organs involved, severity of the disease and biochemical indications.

Although WD is an established monogenic disorder, heterogeneity in phenotype is observed even among patients harboring mutations in *ATP7B *that would affect the mutant protein similarly (e.g. different truncation mutations in the same region of the gene or exactly same set of mutations in two sibs); no particular phenotype can be attributed to a particular mutation [5]. Also, some patients show symptoms atypical of WD [6]. Such observations led to the speculation that there might be modifying loci, which modulate the phenotype resulting from the aberration in the *ATP7B*. One would expect that genes for proteins interacting either directly with ATP7B or influencing it indirectly might fit the role of modifier locus and genes of copper homeostasis pathway could have such potential.

One of the candidate genes which might play the role of a modifier locus is *MURR1 *(renamed *COMMD1*). In dogs, especially in Bedlington Terriers, mutation in *COMMD1 *causes canine toxicosis with symptoms of high hepatic copper accumulation leading to hepatitis and cirrhosis and eventual death between 2 and 6 years of age, unless treated [7]. The causal founder mutation is a deletion of exon 2 resulting in degradation of the protein [8]. Human *COMMD1 *located on chromosome 2 (2p13-p16) codes for a soluble 21-kDa protein, which is 88% identical to its canine orthologue [9]. Though the exact cellular role of COMMD1 is still uncharacterized, it seems that it functions as a cytosolic as well as membrane associated adapter protein linking lipids with other proteins. It specifically binds phosphatidylinositol 4,5-bisphosphate (PtdIns(4,5)P2) through its C-terminal domain. In native conditions, endogenous COMMD1 forms large oligomeric complexes both in the cytosol and at the membrane; interaction with PtdIns(4,5)P2 increases the stability of oligomers [9]. It has been also reported that COMMD1 interacts with the N-terminal domain of ATP7B [10] and was shown to bind copper *in vitro *through residues 61-154 (coded by exon 2) [11]. Knockout of COMMD1 by siRNA leads to elevated copper retention in HEK293 [12]. Interestingly, XIAP (X-linked Inhibitor of Apoptosis) has been observed to negatively regulate COMMD1 protein levels by the formation of K48 polyubiquitin chains on COMMD1 that promote its degradation [12]. Recently, COMMD1 has been identified as an inhibitor of NF-κB signaling pathway [13]. Similar to many other proteins involved in NF-κB signaling, COMMD1 also shuttles between cytoplasm and nucleus. Two highly conserved Nuclear Export Signals (NES) contained within amino acid 121-187 were identified in COMMD1 which are essential and sufficient for its nuclear export. Mutations in the NES region caused nuclear accumulation of COMMD1 and hence inhibition of NF-κB activity [14]. Besides controlling NF-κB activity, COMMD1 also interacts with HIF-1α and inhibits its activity [15].

Close resemblance of copper toxicosis symptoms to Wilson disease led to the idea of examining the role of *COMMD1 *in WD patients particularly with atypical phenotypes [16-18]. Stuehler et al. found a correlation of early age of onset in patients harboring H1069Q homozygous mutation in *ATP7B *with the silent nucleotide change c.492T>C (D164D) in *COMMD1 *[19]. However in a study involving a heterogeneous cohort consisting of Eastern European and Mediterranean WD patients, no correlation was found between the change c.492T>C and the age of disease onset. They also detected the change in homozygous condition among normal individuals [20]. RNA studies to detect possible alteration of splice site, revealed no changes in RNA size for this change [20]. In a study involving Chinese Wilson disease patients, the particular nucleotide change could not be detected [17]. To date, studies involving *COMMD1 *screening in Wilson disease has failed to show any clear correlation between any nucleotide change and a disease phenotype. To identify whether any biochemical change in WD correlates with any change in *COMMD1 *or whether COMMD1 is mutated in atypical Wilson disease patients, we screened the three exons and a large part of the exon-intron boundaries of *COMMD1 *in 109 patients.

## Materials and methods

Wilson disease patients, mostly with neurological problems (106 out of 109), were examined at the Movement Disorders Clinic, Bangur Institute of Neurosciences and Psychiatry, Kolkata, India with referrals being made to the Regional Institute of Ophthalmology, Kolkata and the Gastroenterology and Pediatrics Departments, SSKM Hospital, Kolkata, for ophthalmologic and hepatic cases, respectively. The diagnosis was based on (a) clinically, on the presence of neurological features like dystonia, tremor, rigidity and bradykinesia and/or hepatic features like signs and symptoms of acute or chronic liver failure, cirrhosis of liver etc., and Kayser-Fleischer (K-F) ring in the cornea; and (b) biochemically, on low serum ceruloplasmin (<20 mg/dl) and high 24-h urinary copper excretion (>100 μg) [2], and on CT and MRI scanning where applicable. Diagnosis was based on the entire profile, and presence or absence of any one feature did not contribute towards either diagnosis or exclusion thereof. Since most of the patients are neurologic, liver biopsy was not carried out as a diagnostic measure. On the basis of microsatellite marker and mutation analysis, we identified eight pedigrees where the younger sib was presymptomatic.

We collected blood samples from 109 families that included at least one patient in each family. Of all the patients, ~95% of them had Kayser-Fleischer ring. The internal review committee on research using human subjects cleared the project after proper review as per the regulations of the Indian Council of Medical Research. Approximately 10.0 ml peripheral blood samples were collected with informed consent of WD patients and their family members. Genomic DNA was prepared from fresh whole blood using the conventional phenol-chloroform method or salting-out method, followed by ethanol precipitation after which the DNA was dissolved in TE (10 mM Tris- HCl, 0.1 mM EDTA, pH 8.0). PCR was done to amplify the exons and flanking regions of the *COMMD1 *gene from the DNA of patients using primers described in Table 1. PCR was carried out for each fragment in a total volume of 25.0 μl as described above using 1.5-2.5 mM of MgCl_2 _(as appropriate) with 35 cycles of denaturation at 94°C (30 s), appropriate annealing temperature (varying between 50°C and 60°C) for 45-60 s and an extension temperature of 72°C (60-90 s). Bi-directional sequencing of the PCR products with the same set of primers was done using an ABI 3100 Avant DNA sequencer with dye-termination chemistry. Nucleotide changes were detected by comparing the sequence obtained in the chromatogram with the normal gene sequence (NT_022184; Homo sapiens chromosome 2 genomic contig) using Pairwise BLAST [21].

**Table 1:** The PCR primer sequences for scanning *COMMD1 *gene in WD patients

*Exon*	Forward primer (5' - 3')	Reverse primer (5' - 3')	Amplicon size (bp)
1	GTGGTGGTTTTGCACAGGC	TCCAAGCCGGAGACTACAG	301
2	TTCAGTGATTTAAGAGTCACTC	GAATAGACAAGCTAACATGTAG	456
3	GAGTTTGGTCATGCCAGATG	GTGAGAACCTCTGCACTGG	379

All these PCR primers were also used for sequencing *COMMD1*.

## Results and discussion

**Figure 1:**
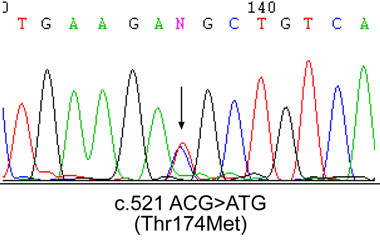
**Electropherogram showing the position of a novel putative mutation c.521 ACG>ATG (Thr174Met) in *COMMD1***. The altered base (C>T) has been marked by an arrow. The nucleotide change and corresponding alteration in the amino acid are indicated at the bottom of the panel.

Three nucleotide changes were identified in *COMMD1*, of which two represented reported synonymous changes viz. c.492 GAT>GAC (Asp164Asp) [19] and a novel variant c.170+122C>T (IVS1+122C>T). The third one a nonsynonymous change (c.521 ACG>ATG; Thr174Met), also represented a novel variant. The chromatogram showing the change c.521 ACG>ATG is furnished in Fig 1. Though the intronic variant appears to be inconsequential, the silent cSNP has been previously reported to be associated with early onset WD phenotype [19]. Hence our observations for both the coding sequence changes are described below:

### c.492 GAT>GAC (Asp164Asp)

We detected the change in two patients in heterozygous condition. One patient was a compound heterozygote for the *ATP7B *mutations c.2495_2496insG and c.2298_2299insC, while *ATP7B *mutations in the other patient are not characterized yet. Available phenotype data for both the patients are furnished in Table 2. It was interesting to note that age of onset (12 years) and the ceruloplasmin levels (5.0 and 5.5 mg/dl) of these two patients were strikingly similar. In addition, both the patients showed marked extrapyramidal features of dystonia, dysarthia and tremor; tremor was localized in the upper limbs in both the patients. However, we have not yet been able to establish a causal relationship of the *COMMD1 *variant with specific disease phenotype since we found the variant in two patients only.

**Table 2:** Genotype-phenotype correlation in patients heterozygous for the *COMMD1 *change c.492 GAT/GAC

Features	Patient W112	Patient W311
GENOTYPIC		
*COMMD1*	c.492 GAT/GAC (heterozygous)	c.492 GAT/GAC (heterozygous)
*ATP7B*	c.2495_2496insG/c.2298_2299insC	Not determined
PHENOTYPIC		
Age of onset (yrs)	12	12
KF ring	+	+
Liver involvement	History of jaundice	Not apparent
Ceruloplasmin	5 mg/dl	5.5 mg/dl
24 hrs Urinary copper	98 μg	170 μg
Extrapyramidal features	Dystonia, dysarthia, tremor in upper limbs	
Liver function test (albumin:globulin; normal:1.7-1.9)	0.85	1.4

### c.521 ACG>ATG (Thr174Met)

The nucleotide variant identified in a single WD patient represents a novel change that occurred in exon 3 of *COMMD1*. The change represented a nonsynonymous mutation, non-conservative in nature (i.e. polar to a non-polar residue). Evolutionary conservation of the residue (Thr) in Murr1 homologue was examined in different species and it was found that like human, chimpanzee has Threonine at the 174^th ^amino acid position whereas in cattle and dog it is the polar Lysine residue. Additionally, the change resides in the region recently identified as the second 'Nuclear Export Signal (NES)' i.e., amino acids 168-179 [14]. Screening the ATP7B gene revealed that the patient harbored the Wilson disease causing mutations c.813TGC>TGA (Cys271Stop) and c.2298_2299insC (Pro767Pro-fs), both resulting in nonfunctional prematurely truncated protein. It is noteworthy to mention that the father of the proband harbored the COMMD1-mutation in heterozygous condition and had normal levels of biochemical parameters and exhibited no signs or symptoms of the disease.

#### Signs and symptoms exhibited by the patient

The WD patient (W297, male, 9yr 6 months) was presented to the clinic with (a) difficulty in speaking and slurring of speech, (b) drooling of saliva during laughing and sleeping, and (c) abnormal posturing of all the four limbs during daily routine activities (e.g. taking food, walking and running). The patient had high neurological predominance and mild hepatic symptoms. At eight years of age he gradually developed slurring of speech and extrapyramidal features like dystonia. The defects were progressive in nature. His higher mental functions were affected and he exhibited intellectual deterioration with failing grades in school. Other complaints included focal jerky involuntary movements during sleep and he had difficulty in writing due to pain. KF ring was prominent in both the eyes. The serum ceruloplasmin of the patient was 7.0 mg/dl (normally >20 mg/dl) that further reduced to 2.5 mg/dl even after administration of the d-penicillamine for two months. It is possibly due to delayed response of the patient to chelation therapy.

We thought that two observations, as described below, were striking in this patient, not common in the WD, despite the fact that the patient responded well on treatment with d-Penicillamine and his conditions improvement with reduction in dystonic features.

(i) The 24-hours urinary copper was excessively high - 1436 μg (normally <100 μg), which reduced to 880 μg after six months of therapy. In our patient pool the 24 hours urinary copper level ranges from 90 - 380 μg. In seven of these patients, 24-hour urinary copper levels ranged between 90-100 μg.

(ii) The clinical record of the patient shows that his two lower incisor teeth were seen within seven days of birth.

In the present scenario involving the Wilson disease patient with the putative COMMD1 mutation Thr174Met (in addition to the *ATP7B *mutations), we speculate that additional copper accumulation over the general WD limits is due to the presence of one mutant allele of *COMMD1*. This might have aggravated copper accumulation is reflected by excessive level of urinary copper.

Interestingly, Wilson disease patients do not exhibit overt clinical features indicating apoptosis. It has been reported that high copper accumulation increases XIAP-Cu interaction, eventually leading to degradation of XIAP [22]. It has also been shown that XIAP binds to COMMD1 and regulates its cellular levels by functioning as an E3 ubiquitin ligase for this protein and promoting its proteasomal degradation [12]. Based on these reports we speculate that the putative mutation (Thr174Met) in COMMD1 caused increased copper accumulation and hence degradation of XIAP which in turn decreased the apoptotic threshold in the patient, upregulating Caspase-3 mediated apoptosis. Dental apoptosis seems to be caspase dependent and Caspase-3 has been shown to be activated during dental apoptosis [23]. In our patient, it is likely that lowering of apoptotic threshold and activation of Caspase-3 might have played an important role in premature development of teeth. However, in this context, it is important to mention that Bedlington Terriers with homozygous knockout of COMMD1 leading to copper toxicosis are not reported to have any defect in dentition. Therefore, to decipher underlying molecular events, appropriate experiments need to be done to examine altered apoptosis, if any, by the *COMMD1 *mutation and involvement of Caspase-3 in the process. It is also likely that, since COMMD1 is reported to influence other cellular pathways involving NF-κB and HIF-1α, the mutant protein might exert its effect by altering these cellular events.

To date this is the first mutation to be reported in *COMMD1 *which appears to influence pathogenesis of the patient involving elevated copper accumulation. It is interesting to note that despite multiple investigations to identify mutations in *COMMD1*, not a single disease causal mutation has been so far identified in this gene. It is likely that, mutation in COMMD1, by itself is not sufficient to cause a disease with a clinical manifestation. Alternatively, since COMMD1 is involved in multiple physiological processes, mutant COMMD1 on both the alleles might cause pre-natal fatality.

In the present case, the occurrence of the *COMMD1 *mutation cosegregating with the compound heterozygous ATP7B mutations appears to have enhanced Wilson disease phenotype. However, it is worth mentioning here that out of 109 patients studied, we did not find any other nucleotide variants in COMMD1 worthy to be labeled as putative mutation. This observation, along with other similar studies, suggests that the COMMD1 variants do not contribute to great phenotypic heterogeneity observed in WD.

## Limitations

Functional evidence for the role of the putative COMMD1 mutation in increased urinary copper as well as altered apoptosis in the WD patient would further support the study.

## Conclusions

In this study, we report a single novel, putative mutation in *COMMD1 *in one WD patient with atypical features. The absence of any other prospective mutation in the gene among 108 patients suggests that *COMMD1 *variants do not have any major contribution towards phenotypic heterogeneity observed in WD.

## Competing interests

The authors declare that they have no competing interests.

## Authors' contributions

AG and IC carried out mutation screening in *ATP7B *and *COMMD1 *genes in most of the patient DNA samples and analysis of the data. In addition, AG was primarily responsible for writing the manuscript. Both SM and MS carried genetic screening of a fraction of the samples for *ATP7B *gene. MS also provided help in drafting the manuscript. SKD examined the WD patients and provided their blood samples and clinical data. KR is the principal investigator of the study responsible for its planning, execution and final draft of the manuscript. All authors read and approved the final manuscript.
